# Corrosion-Fatigue Crack Growth in Plates: A Model Based on the Paris Law

**DOI:** 10.3390/ma10040439

**Published:** 2017-04-22

**Authors:** Jesús Toribio, Juan-Carlos Matos, Beatriz González

**Affiliations:** Fracture & Structural Integrity Research Group (FSIRG), University of Salamanca (USAL), E.P.S., Campus Viriato, Avda. Requejo 33, 49022 Zamora, Spain; jcmatos@usal.es (J.-C.M.); bgonzalez@usal.es (B.G.)

**Keywords:** 316L stainless steel, finite-thickness cracked plate, corner crack, numerical modeling, corrosion-fatigue, fatigue crack propagation, *preferential propagation path*

## Abstract

In this paper, a Paris law-based model is presented whereby crack propagation occurs under cyclic loading in air (*fatigue*) and in an aggressive environment (*corrosion-fatigue*) for the case of corner cracks (with a wide range of aspect ratios in the matter of the initial cracks) in finite-thickness plates of 316L austenitic stainless steel subjected to tension, bending, or combined (tension + bending) loading. Results show that the cracks tend during their growth towards a *preferential propagation path*, exhibiting aspect ratios slightly lower than unity only for the case of very shallow cracks, and diminishing as the crack grows (increasing the relative crack depth)—more intensely in the case of bending than in the case of tension (the mixed loading tension/bending representing an intermediate case). In addition, the crack aspect ratios during fatigue propagation evolution are lower in fatigue (in air) than in corrosion-fatigue (in aggressive environment).

## 1. Introduction

The 316L stainless steel is a structural material widely used in engineering, in particular in the nuclear industry (vessels for fission and fusion reactors), usually immersed in a very aggressive environment and subjected to fatigue (cyclic loading). In this framework, understanding its fatigue and corrosion-fatigue behavior is of the highest importance. Molybdenum content for austenitic stainless steel, tempering temperature for 13% chromium stainless steel, and volume percent ferrite for duplex stainless steel are metallurgical factors that contribute to corrosion-fatigue strength improvement. These factors are strongly involved in corrosion pit formation and in corrosion-fatigue crack initiation processes [1]. The change in the fatigue behavior of 316L stainless steel weldments is frequently related to microstructural modifications [2]. Bulk deformation by rotary swaging and surface deformation by shot peening improve fatigue life in air and in Ringer’s solution [3]. The rotary swaged material improved corrosion resistance, while shot peening led to lower corrosion resistance [3]; however, increasing the shot peening time also produces an improvement in the resistance to pitting corrosion [4].

The existence of free surfaces influences crack propagation, so that although any crack tends to propagate producing a similar stress intensity factor (SIF *K*) along its front (*iso-K propagation*), the presence of the free surface impedes this [5]. Thus, a quarter-elliptical corner crack grows faster than a semi-elliptical surface crack, which grows faster than an elliptical embedded crack, all other conditions being the same [6]. Under tensile loading, corner cracks in plates exhibit higher *K-*values in those points over the crack front near the free surface and lower *K*-values at the inner points. With regard to the crack aspect ratio evaluated as the ratio of the crack depth to the other dimension of the ellipse, the maximum normalized SIFs occurred at the free surface along the width of cracks with aspect ratios greater than 1 and at the free surface along the thickness of those with aspect ratios less than 1 [7], where the ratio of a specimen’s width to its thickness varied from a square bar to a very wide plate (the thickness being the smaller dimension and the width the bigger one). For out-of-plane bending loading, the highest value of the normalized SIF occurred at either end of a shallow crack; the location depending on the crack shape. However, for deep cracks, the highest values occurred at the free surface in the width direction [7]. For corner cracks emerging from a semicircular notch or a through-the-thickness hole, the SIF rises (for the same crack configuration) with the increment of the ratio of the notch (or the hole) radius to the plate thickness [8,9]. Surface cracks growing by fatigue (cyclic) loading tend to a *preferential propagation path* [10], which is different depending on the type of loading and the exponent of the Paris law of the material (cf. [10]). For corner cracks in finite-thickness plates under uniform tensile remote loading, the crack propagation angles along the deflected and inclined crack fronts were shown to increase in magnitude along the whole crack front with increasing deflection or inclination angle [11].

In this paper, a numerical procedure is proposed to calculate the evolution of the crack front (including aspect ratio) in the matter of corner cracks in plates, with special emphasis on the *preferential propagation paths*. The analysis covers propagation both in inert environments (*fatigue in air*) and in aggressive atmospheres (*corrosion-fatigue*).

## 2. Numerical Procedure

**Figure 1:**
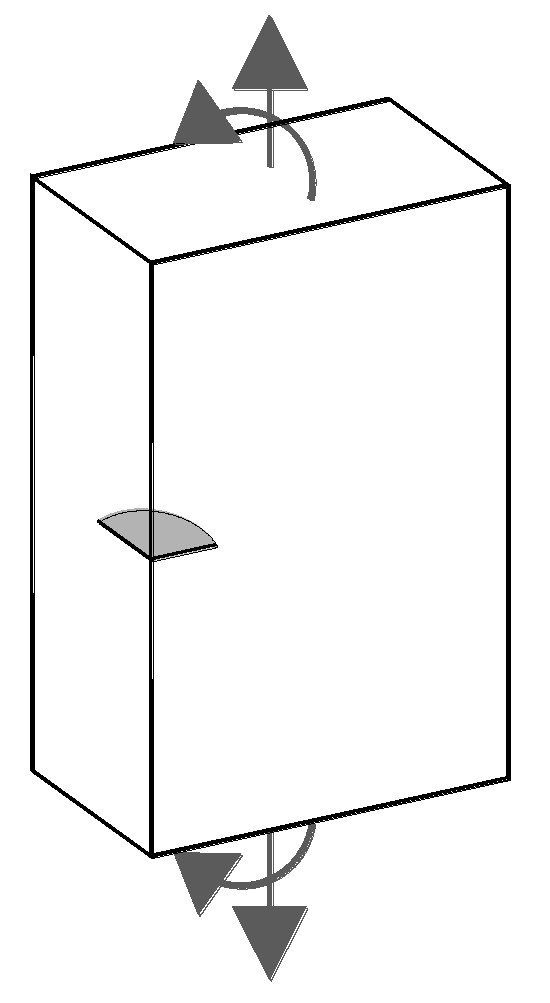
Corner crack in a plate subject to tension and bending.

A numerical modeling using Java language programming is performed to analyze the cyclic propagation paths for corner cracks in plates (of finite thickness) of 316L austenitic stainless steel subjected to fatigue and corrosion-fatigue, the cyclic loading being tensile, bending-type, or mixed (tension + bending), as shown in Figure 1.

### 2.1. Material

The analyzed material is 316L austenitic stainless steel and its fatigue behavior (at room temperature, under sine wave loading conditions, with a stress ratio *R* = 0.05, and frequency of 5 Hz) was characterized by means of the Paris law [12]. In the case of fatigue in air, the cyclic crack growth rate *da*/*dN* is related to the SIF range Δ*K* through the following equation [13]:

(1) $$\frac{da}{dN}=3.61\times 10^{-14}\Delta K^{4.47},$$

which is obtained using compact tension specimen (CTS) in which the dimensions were modified slightly from standard ones to include a greater height-to-width ratio.

Corrosion-fatigue effects due to aqueous saline conditions (in neutral aerated Ringer’s solution: 2.25 mL of NaCl, 0.105 mL of KCl, 0.12 mL of CaCl, and 0.05 ml of NaHCO_3_ for each liter of solution) alter the Paris crack growth law, so that the Paris-type steady state crack growth rate is given by [13]

(2) $$\frac{da}{dN}=8.47\times 10^{-11}\Delta K^{2.23},$$

obtained using the same specimens as in the air tests, as well as the same testing conditions (*R* = 0.05, frequency = 5 Hz).

**Figure 2:**
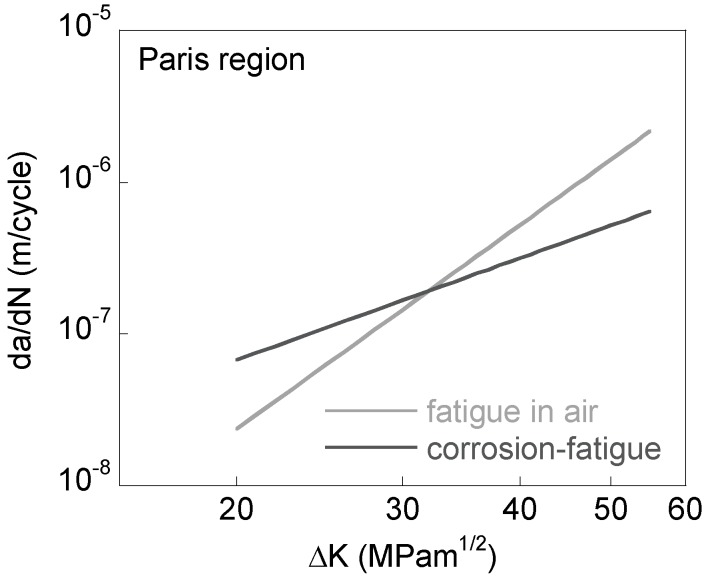
Fatigue crack growth rate as a function of SIF range (Paris region).

It is seen that the crack growth rates in Ringer’s solution are faster than those in air at low SIF ranges. However, this situation is reversed at higher SIF ranges for which crack growth rates in Ringer’s solution actually become slower than in air (Figure 2).

### 2.2. Stress Intensity Factors

**Figure 3:**
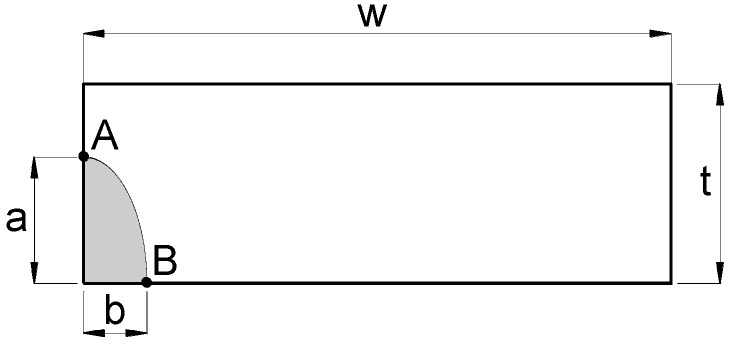
Section of the plate containing the crack. A and B: crack front points intersecting the plate free surface; *a*: crack depth; *b*: crack second dimension; *t*: plate thickness; *w*: plate width.

**Figure 4:**
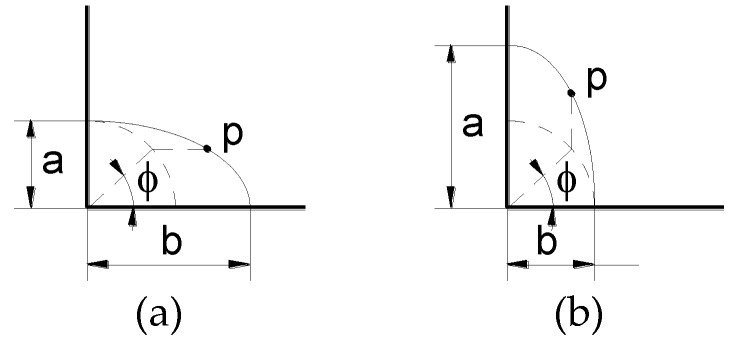
Angle *ϕ* defining a point *p* at the crack front: (**a**) *a*/*b* ≤ 1; (**b**) *a*/*b* > 1. *a*: crack depth;*b*: crack second dimension.

The crack front is characterized as a quarter of an ellipse of semiaxes *a* (crack depth) and *b* (crack second parameter) in a plate of width *w* and thickness *t* (where *t* < *w*; *t*/*w* < 1) (Figure 3). Each point *p* at the crack front is determined by the angle parameter *ϕ* that depends on the ratio between the dimensions *a* and *b* of the ellipse (Figure 4).

The SIFs *K* used in this study were those obtained by Newman and Raju [14] by using 3D finite element analysis and the nodal force method. For a plate subjected to remote tensile stress *σ*_t_ plus bending stress *σ*_b_, containing a quarter-ellipse crack emerging from a corner, these authors fitted their results to the following equation (valid for 0.2 ≤ *a*/*b* ≤ 2, *a*/*t* < 1, *b*/*w* < 0.5, and 0 ≤ *ϕ* ≤ π/2; the ratio *t*/*w* is implicitly given on the basis of the values *a*/*b*, *a*/*t*, and *b*/*w*):

(3) $$K=\left(\sigma_{t}+H_{c}\sigma_{b}\right)\sqrt{\frac{\pi a}{Q}}F_{c}.$$

The function *Q* is the shape factor for an ellipse and is given by the square of the complete elliptic integral of the second class, a function of the aspect ratio *a*/*b*. The functions *F*_c_ and *H*_c_ depend on the following parameters: crack aspect ratio *a*/*b*, relative crack depth *a*/*t*, finite width *b*/*w*, and angle coordinate *ϕ*. The expressions for the calculation of these functions are given in [14].

Although the SIF depends on the specific point on the crack line (defined by the angle *ϕ)*, the finite-width correction factor of the SIF solution provided by Newman and Raju [14] is independent of the angle *ϕ*, so the results expressed in the form of crack front evolutions do not depend on the ratio of plate thickness to plate width (*t*/*w*), thereby providing generality to the results.

### 2.3. Numerical Modelling

The basic hypothesis of this modeling consists of assuming that the crack front can be characterized as an ellipse with its center at the plate corner and that the crack propagation by fatigue is perpendicular to such a front following a Paris law [12]:

(4) $$\frac{da}{dN}=C\Delta K^{m}.$$

To discretize the elliptical crack front, it was divided into *z* segments of the same length, using the Simpson rule. Later, each point *i* at the crack front was shifted (as described in the previous paragraph), keeping constant the maximum crack increment Δ*a*_max_ ≡ max Δ*a_i_* associated with the point of maximum SIF (Δ*K*_max_). On the basis of this maximum increment Δ*a*_max_ and the SIF range Δ*K*, the advance at any point of the crack front Δ*a_i_* is given by:

(5) $$\Delta a_{i}=\Delta a_{\max}{\left(\frac{\Delta K_{i}}{\Delta K_{\max}}\right)}^{m}.$$

Using the least-square method, a new ellipse is fitted to the points of the advanced crack. The process is iteratively repeated up to reaching a relative crack depth *a*/*t* = 1. In addition, a convergence study was made to determine the optimum value of the parameters *z* and Δ*a*_max_, obtaining *z* = 12 and Δ*a*_max_ = 0.000005*t* as the best values.

The proposed method uses the SIF solution thoroughly at any point of the crack, thereby providing better results than those computational methods based *only* on the SIF solutions at the two points at which the crack intersects the free surfaces, as demonstrated in a previous research work [10] in which the crack growth evolution is calculated for surface cracks in plates.

The described numerical propagation model is also applicable in the case of corrosion-fatigue crack initiation from the corrosion pit in aggressive environments, because any pit can be considered as a three-dimensional (3D) defect whose section with the plane of analysis provides the model with an initial geometry of the crack.

## 3. Numerical Results

**Figure 5:**
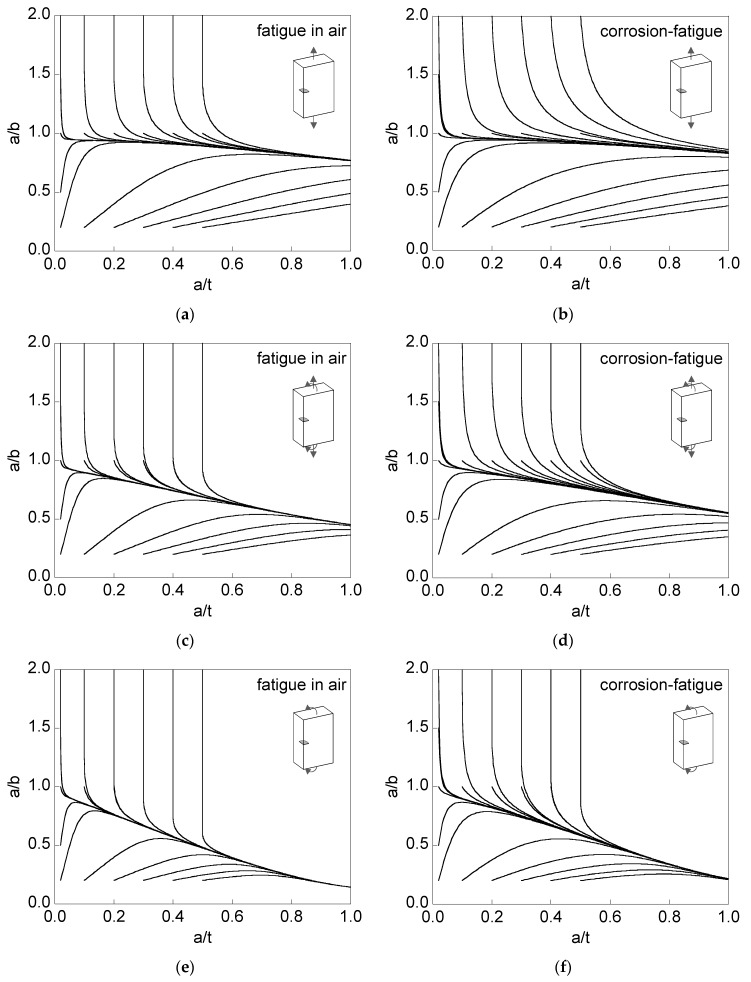
Evolution of the crack aspect ratio in 316L stainless steel: (**a**) fatigue in air and tension; (**b**) corrosion-fatigue and tension; (**c**) fatigue in air and tension + bending; (**d**) corrosion-fatigue and tension + bending; (**e**) fatigue in air and bending; (**f**) corrosion-fatigue and bending.

Figure 5 shows the crack propagation paths (aspect ratio *a*/*b* versus relative crack depth *a*/*t*) for corner cracks, with different initial geometries ((*a*/*t*)_0_, (*a*/*b*)_0_), in finite-thickness plates under fatigue in an inert environment (air) or corrosion-fatigue in an aggressive environment (a saline solution).

The initial geometries used in the modeling were as follows:

(i) (*a*/*t*)_0_ = {0.02, 0.1, 0.2, 0.3, 0.4, 0.5};
(ii) (*a*/*b*)_0_ = {0.2, 0.5, 1.0, 1.5, 2.0} for (*a*/*t*)_0_ = 0.02;
(iii) (*a*/*b*)_0_ = {0.2, 1.0, 2.0} for (*a*/*t*)_0_ = {0.1, 0.2, 0.3, 0.4, 0.5}.

The loading types were as follows:

(i) tension loading *σ*_t_ = *σ*;
(ii) tension loading plus bending moment *σ*_t_ = *σ*_b_ = *σ*/2;
(iii) bending moment *σ*_b_ = *σ*.

It can be observed that the crack propagation curves *a/b-a/t* associated with different initial geometries tend towards a *preferential propagation path*, which is different depending on the case of analysis (environment and loading type). In addition, the convergence (closeness between curves) to such a preferential path is quicker in air than in aggressive environments, and faster in bending than in tension. In the case of combined loading (tension + bending), the convergence is an intermediate between that for tension and that for bending.

## 4. Discussion

**Figure 6:**
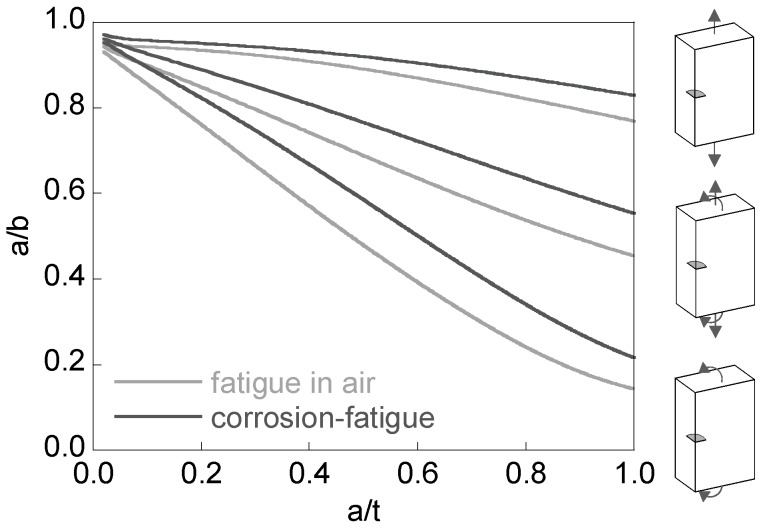
Crack aspect ratio for the preferential propagation path.

Figure 6 shows the curves *a/b-a/t* associated with the preferential cracking paths for fatigue in air and corrosion-fatigue under tension, bending, and combined (tension + bending) loading. The very shallow cracks exhibit an aspect ratio slightly lower than one, and as they propagate (increasing their depth) such an aspect ratio diminishes. This aspect ratio decrease is more pronounced in bending than in tension, the combined loading representing an intermediate case. In corrosion-fatigue (aggressive environment) the Paris exponent is lower, so that the curves related to the preferential crack path are more elevated than those for fatigue in air, i.e., the aspect ratios are higher for the same relative crack depth.

**Figure 7:**
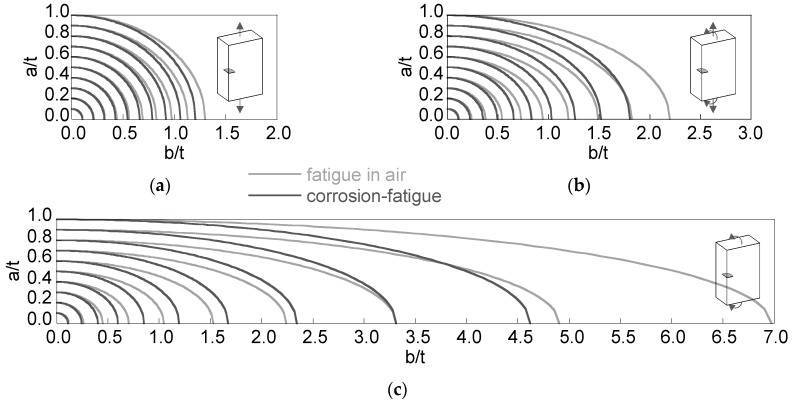
Crack front evolution for the special situation of the *preferential crack propagation path*: (**a**) tension; (**b**) tension + bending; (**c**) bending.

In the case of the *preferential propagation path*, the plots representing the crack fronts for increments of relative crack depth equal to 0.1 (Figure 7) show how the distance between crack fronts for fatigue in air and for corrosion-fatigue increases with the relative crack depth, more intensively in bending than in tension. The *preferential propagation path* for tension + bending exhibits intermediate aspect ratios (between tension and bending), with crack fronts closer to those for tensile loading.

**Figure 8:**
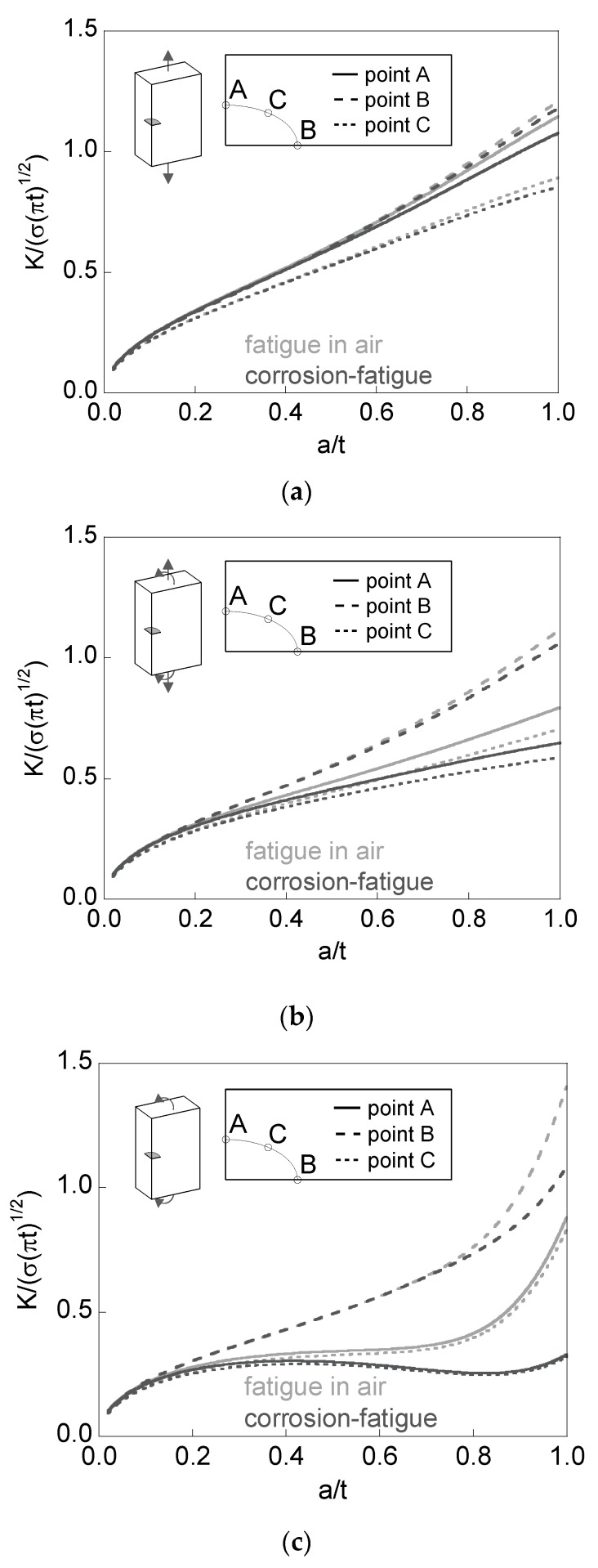
Dimensionless stress intensity factor (SIF) for the special situation of the *preferential crack propagation path*: (**a**) tension; (**b**) tension + bending; (**c**) bending.

Figure 8 plots, for the special case of the *preferential propagation path*, the dimensionless SIF *K*/(*σ*(π*t*)^1/2^) versus the relative crack depth *a*/*t* at the points over the crack front intersecting the free surface of the plate (points A and B in Figure 3), together with the minimum SIF achieved at an intermediate point over the crack front (point C).

For tension loading the dimensionless SIF increases with the relative crack depth, reaching its maximum value for short cracks at point A (almost with the same value as at point B). Such a maximum value is achieved at point B for long cracks under tension. For bending loading, the SIF at point B is (usually) higher than that at point A, whose value is close to the minimum (point C). The difference between the dimensionless SIF values at points A and B increases with the relative crack depth and is higher in bending (with small aspect ratios) than in tension. For mixed loading (tension + bending), the situation is intermediate.

In addition, for plates subjected to bending loading, it can be observed that the dimensionless SIF at points A and C remains constant (or quasi-constant) for fatigue in air during part of the crack growth stage (constant crack growth rate in these points), whereas its value diminishes in corrosion-fatigue (decreasing crack growth rate).

## 5. Conclusions

The following conclusions may be drawn for the fatigue propagation of corner cracks in air and aggressive environments (corrosion-fatigue).

(i) The cracks tend towards a *preferential propagation path*, the convergence being quicker for fatigue in air than for corrosion-fatigue, and faster for bending than for tension, the mixed loading case (tension + bending) representing an intermediate situation.

(ii) The *preferential cracking path* for small relative crack depths exhibits aspect ratios slightly lower than unity, moderately diminishing under tension and markedly under bending as the relative crack depth increases.

(iii) For the *preferential cracking path*, the aspect ratios are higher for corrosion-fatigue than for fatigue in air, the differences between the crack fronts in both phenomena being higher in the case of bending than in the case of tension.

(iv) The *preferential propagation path* for combined (tension + bending) loading exhibits aspect ratios intermediate between those for tension and those for bending, with crack fronts closer to the case of tensile loading.
